# VEGFR-3 and CXCR4 as predictive markers for treatment with fluorouracil, leucovorin plus either oxaliplatin or cisplatin in patients with advanced esophagogastric cancer: a comparative study of the Arbeitsgemeinschaft Internistische Onkologie (AIO)

**DOI:** 10.1186/1471-2407-14-476

**Published:** 2014-07-01

**Authors:** Thomas Thomaidis, Annett Maderer, Salah-Eddin Al-Batran, Janis Kany, Claudia Pauligk, Kristina Steinmetz, Arno Schad, Ralf Hofheinz, Harald Schmalenberg, Nils Homann, Peter Robert Galle, Markus Moehler

**Affiliations:** 1 I. Medical Department, Johannes-Gutenberg University of Mainz, Langenbeckstr.1, 55131 Mainz, Germany; 2 Institute of Clinical- Oncological Research at Krankenhaus Nordwest, UCT University Cancer Center, Frankfurt am Main, Germany; 3 III. Medical Clinic, University Clinic, Mannheim, Germany; 4 Tumor centre, University of Jena, Jena, Germany; 5 II. Medical Clinic, Wolfsburg, Germany

**Keywords:** VEGFR-3, CXCR4, Advanced esophagogastric cancer, FLO, FLP, Biomarkers

## Abstract

**Background:**

Combination of fluoropyrimidines and a platinum derivative are currently standards for systemic chemotherapy in advanced adenocarcinoma of the stomach and gastroesophageal junction (GEJ). Nevertheless, individual likelihood for response to these therapeutic regimes remains uncertain. Even more, no predictive markers are available to determine which patients may benefit more from oxaliplatin versus cisplatin or vice versa. The new invasion and stem cell markers VEGFR-3 and CXCR4 have been linked prognostically with more aggressive esophagogastric cancer types. Thus, we aimed to assess correlations of VEGFR-3 and CXCR4 expression levels with clinical outcome in a randomized phase III study of patients with oxaliplatin/leucovorin/5-FU (FLO) versus cisplatin/leucovorin/5-FU (FLP).

**Methods:**

The patients data examined in this study (n = 72) were from the collective of the FLO vs. FLP phase III AIO trial. Tumour tissues were stained via immunohistochemistry for VEGFR-3 and CXCR4 expression and results were evaluated by two independent, blinded investigators.

Outcome parameter: Survival analysis was calculated for patients receiving FLO vs. FLP in relation to VEGFR-3 and CXCR4 expression.

**Results:**

54% and 36% of the examined tumour tissues showed strong positive expression of VEGFR-3 and CXCR4 respectively. No superiority of each regime was detected in terms of overall survival (OS) in the whole population. Patients with strong expression of CXCR4 on their tumour tissues profited more in terms of OS under the treatment of FLP (mOS: 28 vs 15 months, p = 0.05 respectively). Patients with negative VEGFR-3 and CXCR4 expression had a trend to live longer when FLO regime was applied (mOS: 22 vs. 9 months, p = 0.099 and 20 vs. 10 months, p = 0.073 respectively). In an exploratory analysis of patients older than 60 years at diagnosis, we observed a significant benefit in overall survival for VEGFR-3 and CXCR4-positive patients when treated with FLP (p = 0.002, p = 0.021 respectively).

**Conclusions:**

CXCR4 positive patients profited in terms of OS from FLP, whereas FLO proved to be more effective in CXCR4 and VEGFR-3 negative patients. Our results suggest, despite the limited size of the study, a predictive value of these biomarkers concerning chemotherapy with FLP or FLO in advanced esophagogastric cancer.

## Background

Adenocarcinoma of the stomach and the gastroesophageal junction (GEJ) is one of the most common and lethal malignancies with approximately 990,000 new cases and 738,000 deaths per year worldwide [1]. Most gastric cancers are unfortunately diagnosed at an advanced stage, so that even after a potential curative gastrectomy relapse rates remain at levels of between 40% and 60% [2].

Systemic chemotherapy is nowadays the gold standard for the palliative treatment of patients with advanced or metastatic cancer of the stomach or GEJ. The combination of fluoropyrimidine and platinum is widely considered to be the treatment of choice in advanced gastric cancers having shown a benefit in overall survival (OS) and progression free survival (PFS) in different studies [3-5]. Nevertheless, the focus of recent and current studies remains the identification of a superior treatment combination while minimizing toxicity.

To the best of our knowledge, there are two phase III studies that deal with the effect and toxicity of oxaliplatin compared with cisplatin in the treatment of metastatic esophagogastric cancer [6,7]. Data from the REAL-2 trial [6] showed no inferiority of oxaliplatin versus cisplatin or of capecitabine versus 5-FU for treatment in this category of patients. Moreover in a post-hoc subgroup analysis oxaliplatin proved to be more effective than cisplatin in patients >65 years [7].

In the search of new biomarkers for advanced esophagogastric carcinoma, VEGFR-3 and CXCR4 have recently become the focus of research [8-17]. VEGFR-3 has been associated with lymphangiogenesis, invasion and metastasis of gastric cancer [9,10,18-21] whilst CXCR4 is associated with stimulation of angiogenesis, lymph node metastasis and peritoneal carcinomatosis [16,22-26]. Nevertheless, their role as predictive markers or as potential therapeutic targets in advanced esophagogastric cancer remains unclear. Despite the encouraging results of the addition of bevacizumab in phase II trials in metastatic and loco regional esophagogastric cancer [27,28], a significant benefit in terms of OS was not observed in the phase III AVAGAST trial [29]. Furthermore, there are to date no comparative studies that focus on a correlation of VEGFR-3 and CXCR4 with the clinical outcome using different therapeutic regimes in patients with locally advanced or metastatic adenocarcinoma of the stomach or GEJ.

The aim of this study was to investigate whether VEGFR-3 and CXCR4 could serve as molecular patterns for personalisation of standard chemotherapy in patients with advanced esophagogastric cancer. We therefore examined and compared the effect of combined chemotherapy with oxaliplatin/leucovorin/5-FU (FLO) versus cisplatin/leucovorin/5-FU (FLP) in patients with advanced esophagogastric cancer in relation to tumour VEGFR-3 and CXCR4 expression.

## Methods

### Patients

The patient data examined in this study (n = 72) originate from the collective of the FLO vs. FLP Phase III trial of the AIO. A comparison of the main disease characteristics between patients in our study and the overall trial population is shown in Additional file 1. Patients were recruited in 31 German and one Swiss centre in a time period of 3 years. Eligibility criteria were histological confirmation of locally advanced or metastatic adenocarcinoma of the stomach or GEJ. Patients had to be over 18 years old, have not received any prior palliative chemotherapy, have not suffered from another type of cancer in the previous five years and have a creatinine clearance > 50 ml/min and adequate bone marrow function. Patients gave written informed consent according to the Helsinki protocol before entering the study, which was approved by the ethics committees of the participating institutions and the Federal Institute for Drugs and Medical Devices (Ref. Nr: 4020513).

### Treatment

The participants were randomized into two treatment arms. The FLO-group received infusional oxaliplatin 85 mg/m^2^ and leucovorin 200 mg/m^2^ over 2 hours every 2 weeks, followed by an infusion of 5-FU 2600 mg/m^2^ over 24 hours. Patients in the FLP therapy arm received cisplatin 50 mg/m^2^ every 2 weeks, combined with the weekly infusion of leucovorin 200 mg/m^2^ over 2 hours and FU 2000 mg/m^2^ over 24 hours. After 6 weeks, the FLP-treatment was followed by a 2-week rest period.

### Immunohistochemistry

The expression of VEGFR-3 and CXCR4 was analyzed by immunohistochemistry (IHC). Paraffin-embedded tissue samples were obtained from 70 patients for CXCR4 and 69 for VEGFR-3, due to limited availability of material. The nature of the collected material was mainly tumour resections.

Three micrometer thick tissue sections were cut and mounted on super frost slides. These were deparaffinized, rehydrated and peroxidase blocked (3% H_2_O_2_ in methanol, 30 min). After blocking of nonspecific protein binding sites by using fresh frozen plasma (30 min) slides were incubated with the respective primary antibody VEGFR-3 (sc 321, Santa Cruz Biotechnology, UK, 1:200, 2 h) and CXCR4 (CIO115, Capralogics, USA, 1:300, 1.5 h) at room temperature, as described before [30-32]. Incubation with secondary antibody (anti-rabbit–mouse–goat antibody) was followed by incubation with streptavidin–POD (DAKO, Germany, each 15 min). Specific antibody binding was visualized using DAB solution (DAKO, Germany) and the tissues were counterstained by hemalaun solution (DAKO, Germany). Between each step of staining the specimens were washed in distilled water or DPBS.

Evaluation of staining was performed by two independent, blinded pathologists.

### Statistical analysis

The staining was evaluated by intensity (no staining 0, weak 1, moderate 2, and strong 3) and the extent of the stained tumour area (0% 0, <25% 1, 25-50% 2, 50-75% 3, > 75% 4). These two classifications were added together and divided into the categories negative and positive. Up to a total of 5, staining was scored as negative, from 6 or more it was considered clearly positive. All statistical analyses were done by using MedCalc software 2013 in close cooperation with the IMBEI. The survival analysis was performed by using the Kaplan-Meier method and the log rank test. To investigate the association between the results of immunohistochemistry obtained for VEGFR-3 and CXCR4 and clinical-pathological parameters, univariate statistical analysis were performed using Pearson's Chi-2 test or Fisher's exact test. p values < 0.05 were considered to indicate significant differences.

## Results

### Patients characteristics

**Figure 1:**
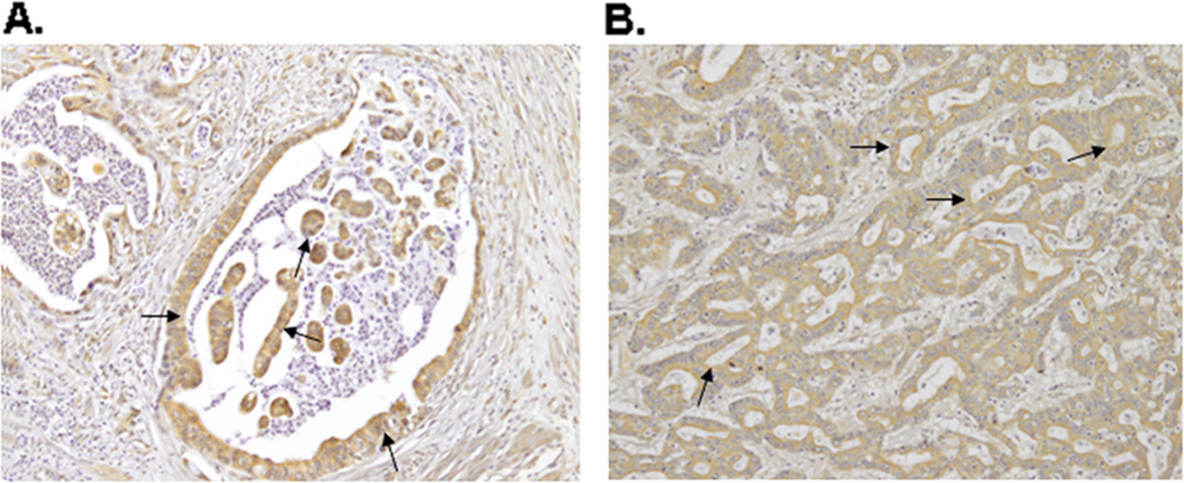
**Immunohistochemistry for VEGFR-3 and CXCR-4 in tumour tissues obtained from patients with esophagogastric adenocarcinoma. A**. strong positive staining (>5) for VEGFR-3, **B**. strong positive staining (>5) for CXCR4 (black arrows).

The average age of patients was 59.8 years and ranged from 33 to 84 years, two thirds of the patients were male. There were no major differences concerning the main disease characteristics in comparison to the overall phase III trial, see Additional file 1. In the statistical analysis of VEGF receptor 3 (VEGFR-3), a total of 69 tumour specimens were included. The cut-off for positive VEGFR-3 expression was drawn at a value of 6, which was true for 39 specimens (54%). 36 patients were treated with FLO, 33 patients received FLP (Table 1). In the statistical analysis of CXCR4, tissue samples from 70 patients were included. 37 of these patients received the FLO regimen, 33 were treated with FLP. The cut-off for a positive detection was set at 6 as sum of the scores for proportion and intensity of staining (Figure 1). In 26 (36%) of all tissue samples, a strong CXCR4 expression could be detected.

**Table 1:** Patient characteristics

**Characteristic**	**All (stained)**		**FLO**		**FLP**		**VEGFR-3 +**		**VEGFR-3 -**		**CXCR4 +**		**CXCR4 -**	
	**n**		**n**		**n**		**n**		**n**		**n**		**n**	
**Number**	72		38		34		39		30		26		44	
**Age (years)**		**SD +/−**		**SD +/−**		**SD +/−**		**SD +/−**		**SD +/−**		**SD +/−**		**SD +/−**
**Mean**	59.8	12.04	59.7	12.76	59.9	11.39	62.4	12.33	55.6	10.8	59	12.6	60	11.95
**Range**	33.1-84.2		33.1-84.2		34.7-78.9		34.7-84.2		33.1-78.9		33.1-84.2		34.7-78.9	
**Gender**		%		%		%		%		%		%		%
**Male**	48	66.7	19	50	29	85.3	26	66.7	19	63.3	19	73.1	28	63.6
**Female**	24	33.3	19	50	5	14.7	13	33.3	11	36.7	7	26.9	16	36.4
**Primary**														
**Cardia**	19	26.4	10	26.3	9	27.3	10	25.6	7	24.1	6	23.1	12	27.9
**Corpus**	27	37.5	15	39.5	12	36.4	14	35.9	12	41.4	12	46.2	15	34.9
**Antrum**	13	18.1	7	18.4	6	18.2	7	17.9	6	20.7	4	15.4	9	20.9
**Esophagus**	2	2.8	1	2.6	1	3	2	5.1	0	0	0	0	1	2.3
**Stomach**	6	8.3	2	5.3	4	12.1	4	10.3	2	6.9	2	7.7	4	9.3
**No information**	4	5.6	3	7.9	1	3	2	5.1	2	6.9	2	7.7	2	4.7

There was an equal distribution of the patients in regards of treatment and positivity of markers (Table 2). Location of the primary and age showed no statistical significant changes within all groups (Table 1).

**Table 2:** Distribution of patients’ treatment in relation to expression of VEGFR-3 or CXCR4

**Characteristic**	**FLO**		**FLP**	
	**n**	**%**	**n**	**%**
**VEGFR-3 +**	22	56.4	17	43.6
**VEGFR-3 -**	14	46.7	16	53.3
**CXCR4 +**	13	50	13	50
**CXCR4 -**	24	54.5	20	45.5

### General statistical analysis

In terms of efficacy, no difference in the response to the treatment regimens FLO and FLP could be detected (p = 0.839). Furthermore, there was no significant difference in survival between VEGFR-3 positive and negative patients or for CXCR4 expression (data not shown).

### Results for the VEGF receptor 3

**Figure 2:**
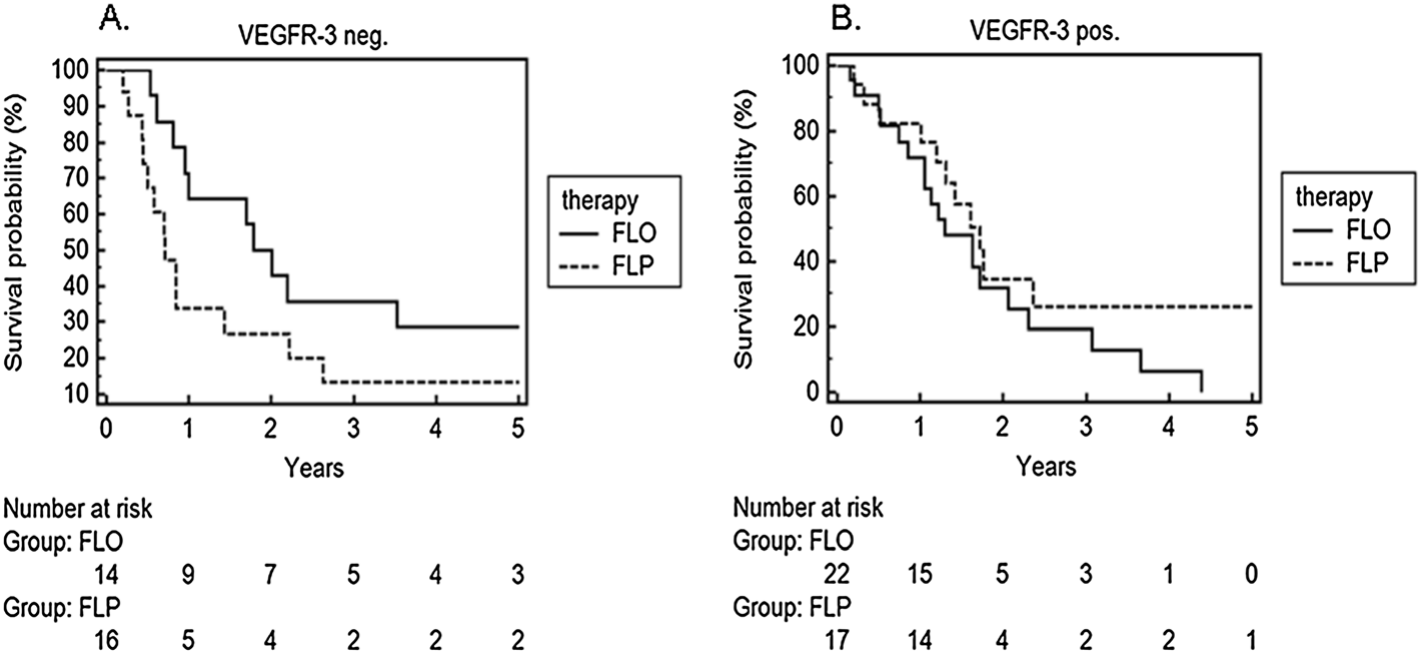
**Survival analysis in patients treated with FLO versus FLP in relation to the expression of VEGFR-3. A**. VEGFR-3 negative in observation time of 5 years. **B**. VEGFR-3 positive in observation time of 60 months.

The response to either therapy regimen strongly depended on the expression status of VEGFR-3. Patients with a negative VEGFR-3 state significantly benefited from treatment with FLO in survival over 18 months (p = 0.026). A strong trend in favour of the FLO-therapy was detected (Figure 2A, p = 0.099), in terms of 5-year survival. The median survival for VEGFR-3 negative patients was 22 months in FLO, compared to 9 months in the FLP arm and was statistically significant. Patients with positive VEGFR-3 status had a median survival benefit of 5 months under FLP treatment (21 months under FLP vs. 16 months under FLO). In the 5-year survival, a trend in favour of the FLP-therapy group was found for patients with strong expression of VEGFR-3 (Figure 2B, p = 0.227).

### Results for the CXC receptor 4

**Figure 3:**
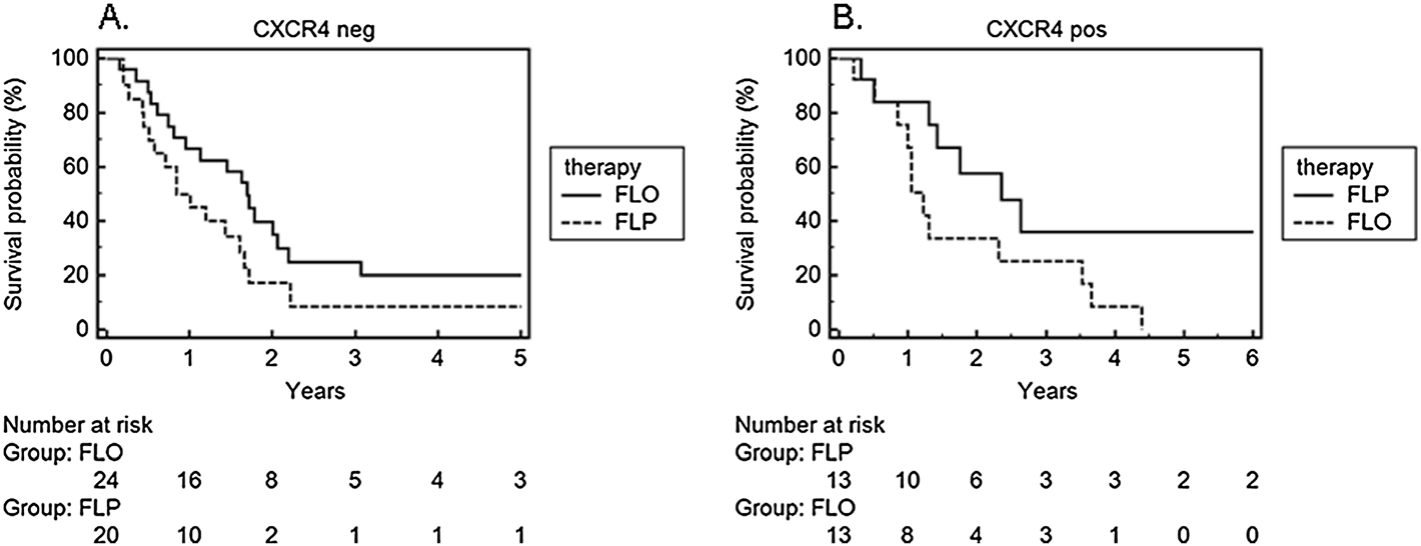
**Survival analysis under FLO/FLP treatment in regards of CXCR4 expression in an observation period of 5 years. A**. CXCR4 negative. **B**. CXCR4 positive.

The CXCR4 expression in tumour tissues showed a strong impact on survival in both treatment groups FLO and FLP. While CXCR4 negative patients showed a trend towards treatment benefit with FLO (Figure 3A, p = 0.073), patients with strong CXCR expression survived longer under FLP treatment (Figure 3B, p = 0.05). The median survival of patients with negative CXCR4-status was a statistic relevant 10 months longer under FLO than FLP (20 vs. 10 months), while strong CXCR4 expression was associated with a statistically significant median survival benefit of 13 months for FLP compared with FLO (28 vs. 15 months, p = 0.05).

### Patients older than 60 years

At this part of our study we performed an exploratory analysis examining whether VEGFR-3 and CXCR4 show the same predictive value for older patients.

**Figure 4:**
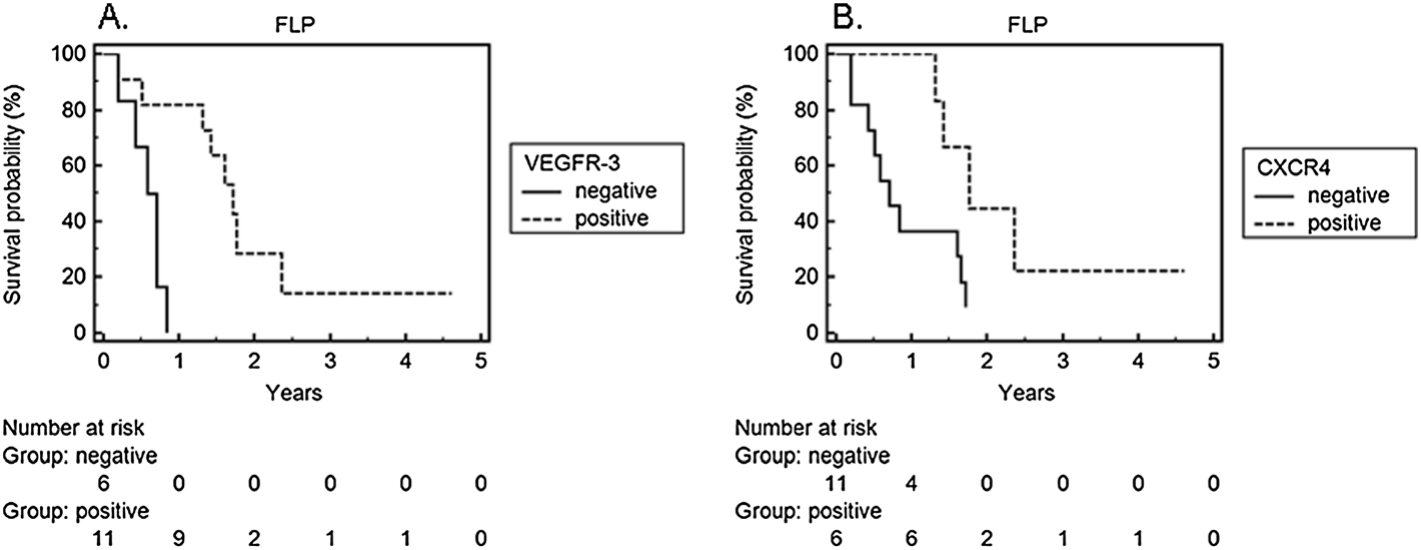
**Survival analysis under FLP treatment for patients older than 60 years. A**. VEGF-R3 and **B**. CXCR4 expression in an observation period of 5 years.

In the post-hoc subgroup of patients older than 60 years at diagnosis, we found a significant benefit in overall survival for VEGFR-3 and CXCR4 positive patients when treated with FLP (Figure 4A and B, p = 0.002, p = 0.021 respectively). No patient of this subgroup with negative VEGFR-3 expression status survived the first year after diagnosis of gastric cancer. However, similar findings were not observed when older patients with weak tumour expression of VEGFR-3 and CXCR4 were treated with FLO (data not shown).

### Combination of the results for VEGFR-3 and CXCR4

**Figure 5:**
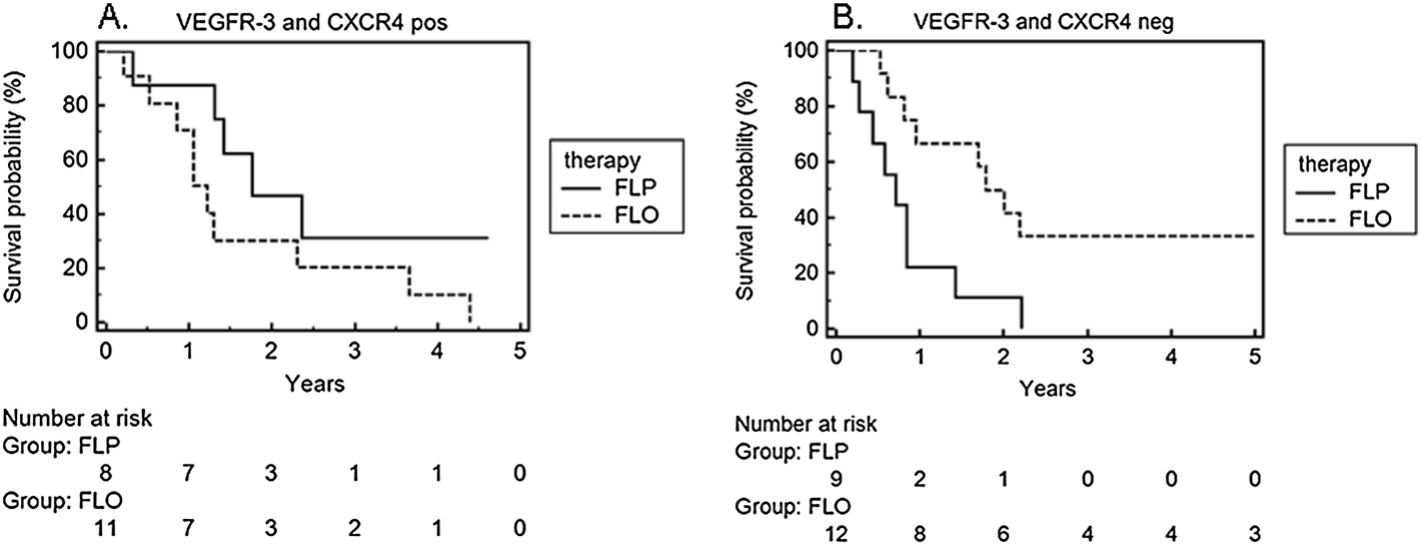
**Survival analysis under FLO/FLP treatment in an observation period of 5 years. A**. VEGF-R3 and CXCR4 positive. **B**. VEGF-R3 and CXCR4 negative.

Patients with a strong expression of VEGFR-3 and CXCR4 benefited in overall survival from the treatment with the cisplatin-containing FLP scheme (Figure 5A, p = 0.126). In contrast, patients with weak expression of CXCR4 and VEGFR-3 lived significantly longer with FLO (Figure 5B, p = 0.011).

## Discussion

Seeking reliable markers to predict chemotherapeutic efficacy in advanced esophagogastric cancer, tumour tissues from 72 patients from the collective of the FLO vs. FLP phase III study of the AIO were tested for expression of VEGFR-3 and CXCR4. Both of these molecules have recently been associated with aggressive and metastatic esophagogastric cancer [8,10,12-14,16,20,22,25,26].

As previously described [6,7], no significant difference between the therapeutic regimes FLO vs FLP was observed in terms of survival of the overall population over 5 years.

In our collective almost half of the specimens showed a strong positivity for VEGFR-3 (54%), whereas 36% of all tissue samples proved to be strongly positive for CXCR4 as assessed by immunohistochemistry. Similar findings have been reported previously in esophageal adenocarcinoma [17,33] as well as for adenocarcinoma of the stomach [11,34]. Taking into consideration that the majority of the CXCR4 and VEGFR-3 measurements, according to the literature, have been performed on tumour tissues after curative gastrectomy, we could expect an increase of the expression of these molecules in patients who are treated in a palliative setting.

In the present study there was a clear benefit in survival when the VEGFR-3 negative patients were treated with oxaliplatin instead of cisplatin. The stronger benefit in this category of patients was seen in the first 18 months of treatment. Conversely, patients with strong VEGFR-3 expression responded better under a cisplatin-containing regimen. Similar results have been reported by Ni et al. [35] who showed significantly longer overall survival when FLO was administered in patients with advanced gastric cancer and low VEGFR-3 serum levels. The median OS was 15.4 months whereas in our study it was 22 months. Ni et al. however, measured only soluble VEGFR-3, thus a direct comparison between these two studies cannot be made. Nevertheless, both of these studies indicate high VEGFR-3 expression is a poor prognostic marker in terms of survival for patients receiving FLO.

Although the correlation of VEGFR-3 expression and response to chemotherapy has been the focus of research in various forms of cancer [36-38], there are only limited data concerning gastric cancer [39,40]. We have previously shown the addition of the VEGFR-1-3 inhibitor, sunitinib to enhance the chemosensitivity of gastric cancer cell lines *in vitro*[39]. Our present results demonstrate a clear benefit of patients with strong VEGFR-3 expression in favour of FLP specifically when compared with patients with a low tumour VEGFR-3 expression (Figure 2). In those patients older than 60 years the benefit of a cisplatin containing therapy was even greater in the VEGFR-3 positive group, as no patient in the VEGFR-3 negative subgroup receiving FLP survived the first year (Figure 4A).

A possible explanation for the enhanced chemosensitivity of VEGFR-3 positive gastric cancer cells to cisplatin may be deduced by inhibition of the Notch pathway. Tamella and colleagues [9] revealed that VEGFR-3 upregulation in endothelial tip cells, caused by inhibition of the Notch signalling pathway, played a crucial role in sprouting angiogenesis in tumours. Since Notch inhibition has also been connected with the enhanced toxicity of cisplatin in colorectal cancer lines and nasopharyngeal adenocarcinoma [41,42], our results may indicate a similar pathophysiological mechanism for advanced esophagogastric cancer. The reason why oxaliplatin appeared to be less effective in VEGFR-3 positive patients remains unknown. However our data are consistent with those of Aleksic et al. [41], who noticed that several colorectal cancer lines showed no responsiveness to oxaliplatin, compared with cisplatin, when Notch signalling was blocked.

When CXCR4 expression levels on tumour tissues were measured similar results were observed. Patients with weak CXCR4 expression profited more from FLO whereas CXCR4 positive patients had a significantly longer 5 year overall survival under FLP (Figure 3). This effect could also be clearly seen in patients older than 60 years with strong CXCR4 positivity as they showed a better response to FLP than the CXCR4 negative ones (Figure 4B).

To date, the significance of CXCR4 as a potential predictive marker for chemotherapy in gastric cancer has been reported only in cellular models [43]. Xie et al. showed a correlation of CXCR4 mRNA levels in gastric cancer with docetaxel sensitivity, whereas the blockade of CXCR4 enhanced docetaxel toxicity. Nevertheless, there are no data that provide a possible explanation for the better responsiveness of CXCR4 positive esophagogastric cancer to cisplatin than to other platinum derives.

It is of great interest in relation to the connection of extracellular-signal related kinases (ERKs) with the stromal cell-derived factor 1 (SDF-1) mediated pathway. Similar to VEGFR-3 [41,44,45], the activation of CXCR4 by its natural ligand SDF-1 leads to phosphorylation and activation of multiple intracellular domains including ERKs [46-49]. Since cisplatin relies on ERK activation for bioactivity in some cells [41,44], in contrast to oxaliplatin [50], this might explain the chemosensitivity of VEGFR-3 and CXCR4 positive esophagogastric adenocarcinoma to FLP and not to FLO.

## Conclusions

To the best of our knowledge this is the first comparative study of FLP and FLO in terms of VEGFR-3 and CXCR4 tumour expression. The list below shows the main findings and conclusions of our trial in accordance to the REMARK guidelines. The main limitation of our study is its size (n = 72) and thus, its power is not very high. However, our results suggest a predictive value of these biomarkers concerning chemotherapy with FLP or FLO in advanced esophagogastric cancer. A trend of longer OS was observed when CXCR4 and VEGFR-3 positive patients were treated with FLP, whereas FLO proved to be more effective in CXCR4 and VEGFR-3 negative patients. Further studies are required in order to investigate the predictive value of VEGFR-3 and CXCR4 in terms of chemotherapeutic regimes in patients with advanced adenocarcinoma of the stomach and GEJ.

List of main steps and findings of the current study according to the REMARK guidelines

Introduction

1. VEGFR-3 and CXCR4 were the examined markers at this study. The objectives of the current study were to examine, whether VEGFR-3 and CXCR4 could have a predictive value in patients with advanced esophagogastric cancer under the treatment of FLO vs. FLO regime.

Materials and Methods

Patients

2. Patients with histologically confirmed locally advanced or metastatic adenocarcinoma of the stomach or the esophagogastric junction were eligible. Patients characteristics, inclusion and exclusion criteria have already been described before [7].

Specimen characteristics and assay methods

3. Paraffin-embedded tissue samples were obtained from patients with advanced esophagogastric cancer. The expression of VEGFR-3 and CXCR4 was analyzed by immunohistochemistry. Evaluation of staining was performed by two independent, blinded investigators.

Study design

4. This study is a retrospective translational analysis of a phase III trial in metastatic gastroesophageal adenocarcinoma. Patients were treated with fluorouracil, leucovorin plus either oxaliplatin or cisplatin as described before [7]. Main end point of the study was overall survival in relation to VEGFR-3/CXCR4 under palliative chemotherapy.

Statistical analysis methods

5. The staining was evaluated by intensity and the extent of the stained tumour area. These two classifications were added together and divided into the categories negative and positive. All statistical analyses were done by using SPSS statistical analysis software. The survival and univariate analysis were performed by using Kaplan-Meier method, log rank test, Pearson's Chi-2 test or Fisher's exact test.

Result

Data

6. The primary tumour location as well as the basic demographic characteristics of the patients who participated at the current trial is demonstrated in Table 1. There was an equal distribution of the patients in regards of treatment and positivity of markers.

Analysis and presentation

7. In the survival analysis, patients with strong expression of CXCR4 on their tumour tissues profited more in terms of overall survival under the treatment of FLP. Patients with negative VEGFR-3 and CXCR4 expression had a trend to live longer when treated with FLO. In an exploratory analysis of patients older than 60 years at diagnosis, there was a significant benefit in overall survival for patients with strong VEGFR-3 and CXCR4 expression when treated with FLP.

Discussion

8. CXCR4 positive patients profited in terms of OS from FLP, whereas FLO proved to be more effective in CXCR4 and VEGFR-3 negative patients. The main limitation of our study is its not very high power, due to the size of the examined tissues (n = 72). However, our results suggest a predictive value of these biomarkers concerning chemotherapy with FLP or FLO in advanced esophagogastric cancer. Further studies are required in order to investigate the predictive value of VEGFR-3 and CXCR4 in terms of chemotherapeutic regimes in patients with advanced adenocarcinoma of the stomach and the gastroesophagic junction.

## Abbreviations

VEGFR-3: Vascular endothelial growth factor receptor 3; CXCR4: CXC-chemokine receptor type 4; GEJ: Gastroesophageal junction; FLO: Oxaliplatin/leucovorin/5-FU; FLP: Cisplatin/leucovorin/5-FU; AIO: Arbeitsgemeinschaft Internistische Onkologie.

## Competing interests

After the last recruited patient M Moehler and S. -E. Al-Batran received honoraria for presentations in satellite symposia by Sanofi-Aventis. The rest of the authors have no competing interests to declare.

## Authors’ contributions

TT: acquisition of data, analysis and interpretation of data, drafting of the manuscript, critical revision of the manuscript, statistical analysis, AM: acquisition of data, analysis and interpretation of data, critical revision of the manuscript, statistical analysis. SEA: study concept and design, patients' recruitment, JK: acquisition of data, analysis and interpretation of data. CP: patient's recruitment, critical revision of the manuscript, KS: patient's recruitment, technical or material support. AS: acquisition of data, RH: patient's recruitment, technical or material support, HS: patient's recruitment, technical or material support, NH: patient's recruitment, technical or material support. PRG: critical revision of the manuscript, administrative support, study supervision. MM: study concept and design, drafting of the manuscript, critical revision of the manuscript, study supervision. All the authors critically reviewed the paper. All authors read and approved the final manuscript.

## Pre-publication history

The pre-publication history for this paper can be accessed here:

http://www.biomedcentral.com/1471-2407/14/476/prepub

## Supplementary Material

Additional file 1 Comparative patient characteristics between the translational study and the overall phase III trial. Click here for file
