# Supplementary material for: VEGFR-3 and CXCR4 as predictive markers for treatment with fluorouracil, leucovorin plus either oxaliplatin or cisplatin in patients with advanced esophagogastric cancer: a comparative study of the Arbeitsgemeinschaft Internistische Onkologie (AIO)
Source: BMC Cancer. 2014 Jul 1;14:476. doi: 10.1186/1471-2407-14-476 (PMC4094395; doi:10.1186/1471-2407-14-476)
Supplement: Additional file 1 — Comparative patient characteristics between the translational study and the overall phase III trial. [file 1471-2407-14-476-S1.doc]

|  | **Translational study** | | | | **Phase III trial** | | | |
| --- | --- | --- | --- | --- | --- | --- | --- | --- |
| **Patient characteristics** | **FLO** | **%** | **FLP** | **%** | **FLO** | **%** | **FLP** | **%** |
|  |  |  |  |  |  |  |  |  |
| Total no. of patients | 38 |  | 34 |  | 112 |  | 108 |  |
|  |  |  |  |  |  |  |  |  |
| Prior surgery: | 25 | 65.8 | 17 | 50 | 51 | 45.6 | 45 | 41.8 |
| Esophagectomy | 1 | 2.6 | 1 | 2.9 | 4 | 3.6 | 2 | 1.9 |
| Total Gastrectomy | 16 | 42.1 | 10 | 29.4 | 33 | 29.5 | 29 | 26.9 |
| Partial Gastrectomy | 8 | 21.1 | 6 | 17.6 | 14 | 12.5 | 14 | 13 |
| ECOG Status: |  |  |  |  |  |  |  |  |
| Median | 1 |  | 1 |  | 1 |  | 1 |  |
| Disease status: |  |  |  |  |  |  |  |  |
| Newly diagnosed | 22 | 57.9 | 25 | 73.5 | 79 | 70.5 | 80 | 74.1 |
| Recurrent | 17 | 44.7 | 8 | 23.5 | 33 | 29.5 | 28 | 25.9 |
| Locally advanced | 0 | 0 | 2 | 5.9 | 3 | 2.7 | 10 | 9.3 |
| Metastatic | 38 | 100 | 31 | 91.2 | 109 | 97.3 | 98 | 90.7 |
